# The effects of helium, strontium, and silver triple ions implanted into SiC

**DOI:** 10.1016/j.heliyon.2023.e20877

**Published:** 2023-10-10

**Authors:** G. Ntshobeni, Z.A.Y. Abdalla, T.F. Mokgadi, M. Mlambo, E.G. Njoroge, M. Msimanga, A. Sohatsky, V.A. Skuratov, T.T. Hlatshwayo

**Affiliations:** a Physics Department, University of Pretoria, Pretoria, South Africa; b ENGAGE, University of Pretoria, Pretoria, 0002, South Africa; c Physics Department, Tshwane University of Technology, P Bag X680, Pretoria, 0001, South Africa; d Joint Institute for Nuclear Research, Dubna, Russia; e Dubna State University, Dubna, Moscow Region, Russia; f National Research Nuclear University MEPhI, Moscow, Russia; g iThemba LABS TAMS, WITS, P Bag 11, 2050, Johannesburg, South Africa; h Health Platform, Advanced Materials Division, Mintek, 200 Malibongwe Drive, Randburg, South Africa

**Keywords:** Implantation, SiC, He-bubbles, Cavities

## Abstract

The effects of helium (He), silver (Ag) and strontium (Sr) ions triple implanted into polycrystalline silicon carbide (SiC) were investigated. Ag ions of 360 keV were first implanted into polycrystalline SiC to a fluence of 2 × 10^16^ cm^−2^ at 600 °C, followed by implantation of Sr ions of 280 keV to a fluence of 2 × 10^16^ cm^−2^ also at 600 °C (Ag + Sr–SiC). Some of Ag + Sr–SiC samples were then implanted with 17 keV He ions to a fluence of 1 × 10 ^17^ cm^−2^ at 350 °C (Ag + Sr + He–SiC). Some of the dual (Ag + Sr–SiC) and triple (Ag + Sr + He–SiC) implanted samples were annealed at 1000 °C for 5 h. Both dual and triple implantation resulted in the accumulation of defects without amorphization of SiC structure. Moreover, triple implantation also resulted in formation of elongated He nano-bubbles and cavities in the damaged SiC accompanied by the appearance of blisters and craters on the surface. Healing of some structural defects was observed after annealing at 1000 °C in both dual and triple implanted samples. Implantation of Sr caused pre-implanted Ag to form precipitates indicating some limited migration while implantation of He caused some migration of both Ag and Sr. The migration of Ag was accompanied by formation of bigger precipitates trapped in He-cavities. Annealing the triple implanted caused the migration of both Ag and Sr governed by trapping of both implanted species by cavities due to some exo-diffusion of He. No migration was observed in the dual implanted samples annealed at 1000 °C. Hence, He bubbles assisted migration of implants and He cavities trap the implanted species.

## 1 Introduction

Containment of radioactive fission products (FPS) is a cornerstone for both safety improvement and the revival of fission nuclear reactors as a clean energy source. In modern generation IV nuclear reactors, safety is enhanced by using a fission products self-retaining fuel particle known as tri-structural isotropic (TRISO) particle [1]. In this self-retaining fuel particle, the kernel is coated with four chemical vapour deposited (CVD) layers of carbon and silicon carbide (SiC). Among these coating layers, SiC is the main diffusion barrier of fission products (FPs). TRISO particles retain quite well the majority of fission products with exception of some radioactive FPs such as silver (^110m^Ag) and strontium (Sr) [2]. ^110m^Ag is a strong gamma emitter with a half-life of 250 days [3]. Thus, if released it can cause danger to personnel and environment. ^90^Sr is a radioactive isotope of strontium produced by nuclear fission. ^90^Sr is one of the most important fission products due to its relatively high yield, uptake and retention in the biological system [4]. It is a high energy beta emitter with a half-life of 28.6 years [5]. Therefore, the release of strontium into the environment could cause a health hazard. It can be deposited in the bones in a similar manner to calcium uptake and can cause cancer of the bones [6,7]. For these reasons, silver and strontium are important FPs that deserve more attention. Several investigations have been conducted on the migration behaviour of Ag and Sr in SiC at high temperatures mimicking the operating and accident conditions of fission nuclear reactors - see Refs. [[8], [9], [10], [11], [12], [13]] and references therein.

During nuclear fission process in a core of the fuel kernel, uranium 235 absorbs a thermal neutron to form uranium 236 which then splits into two fission products (FPs) while releasing two or three neutrons and a lot of energy [14]. Some of the released FPs are unstable or radioactive nuclei and may emit gamma radiation, beta or alpha, or even neutrons, depending on the type of radioactive decay involved, i.e., the manner in which the resulting new nuclei becomes stable. In a nuclear reactor, helium accumulation is possible due to emission of α-particles from nuclear reactions as well as from nuclear transmutation [15,16]. Because of the very low solubility in most solids, and being inert gas, He tends to cluster and form bubbles in SiC, which makes it difficult to migrate [17,18]. These bubbles can induce detrimental modifications of the materials’ structure and mechanical properties [17]. Some studies have reported the effects of He on the microstructural and mechanical properties changes of SiC [15,17]. Moreover, it has also been reported that helium-vacancy clusters form bubbles upon annealing, which causes the properties of SiC to deteriorate [[19], [20], [21]]. Thus, He bubbles may cause SiC to lose its legitimacy as the main diffusion barrier for the fission products in TRISO coated fuel particles.

To fully understand the migration behaviour of different fission products (FPs) in SiC, their migration needs to be investigated in environments mimicking the nuclear environment i.e., where FPs coexist in the presence of helium at elevated temperatures. Limited work has been done on the role of helium in the migration of FPs surrogates such as Ag, Fe, Mg, Kr, and Sr, implanted into SiC [12,[22], [23], [24], [25]]. However, no work has been reported on the influence of helium in the migration of FPs co-implanted into SiC. Such a study is crucial as FPs co-exist in the presence of helium in fission nuclear reactors.

In this study, the effects of helium, strontium and silver triple implanted into SiC were investigated. Ag was first implanted into polycrystalline SiC, the Ag implanted SiC samples were then implanted with Sr ions to the same projected range as Ag (Ag + Sr–SiC). Some of the dual (Ag + Sr–SiC) implanted SiC samples were implanted with He ions to the same projected range as Ag and Sr (Ag + Sr + He–SiC). The dual (Ag + Sr–SiC) and triple implanted (Ag + Sr + He–SiC) samples were then annealed at 1000 °C for 5 h. The structural evolutions and migration behaviour were monitored in both dual and triple implanted SiC before and after annealing. To evaluate the role of He, the results of dual and triple implants were compared.

## 2 Experimental methods

Polycrystalline SiC wafers used in this study were from Valley Design Corporation. The wafers were mainly composed of 3C–SiC with some 6H–SiC present [9,26]. Ag ions of 360 keV were implanted into polycrystalline SiC to a fluence of 2 × 10^16^ cm^−2^ at 600 °C. On the same samples, Sr ions of 280 keV were also implanted to a fluence of 2 × 10^16^ cm^−2^ at 600 °C (Ag + Sr–SiC). Some of the Ag + Sr–SiC samples were then implanted with 17 keV He ions to a fluence of1 × 10 ^17^ cm^−2^ at 350 °C (Ag + Sr + He–SiC). The energies of the implants were chosen such that they have the same projected ranges to allow the synergetic study. Implantation temperatures were maintained above the critical amorphization temperature of SiC to avoid amorphization [26]. The implantation of Ag and Sr was performed at Friedrich-Schiller-Universität Jena in Germany, while the implantation of He was performed at iThemba LABS, Gauteng, South Africa. The flux was kept below 10^13^ cm^−2^ for all implantations.

Both the Ag + Sr–SiC and Ag + Sr + He–SiC samples were annealed at 1000 °C for 5 h in vacuum (10^−4^ Pa), using a computer-controlled *Webb 77* graphite furnace. Transmission electron microscopy (TEM) was performed on the as-implanted Ag + Sr + He–SiC and annealed Ag + Sr + He–SiC samples. Annealing temperature was chosen to mimic the operation temperature of the modern fission nuclear reactor. TEM lamellae were prepared using focussed ion beam (FIB) technique-*FEI Helios Nanolab 650* FIB. Thinning of the samples was achieved by successive 30 keV and 5 keV Ga ions. Finally, polishing was done at 2 keV and 500 eV which produced nearly damage free TEM foils. TEM and energy dispersive X-ray (EDX) elemental mapping were performed using the *Thermoscientific Talos F200i* field emission transmission electron microscope at the Joint Institute for Nuclear Research (JINR), Dubna, the accelaration voltage of 200 kV was used.

Raman spectroscopy (*Witec alpha 300 RAS+*, Germany) was used to characterize the dual and triple implanted samples before and after annealing. During analysis, the laser wavelength of 532 nm, laser power of 20 mW and the 100 × 0.9 numeric aperture objective were used to collect single-spectra using an integration time of 10 s and 10 accumulations. Scanning electron microscopy (SEM) was used to monitor morphological changes of the dual and triple implanted samples before and after annealing using the *Zeiss Gemini Ultra Plus* field emission gun scanning electron microscopy (FEG-SEM) utilizing the in-lens detector mode. During SEM measurements accelerating voltage of 2 kV was used. Moreover, the morphology of the triple implanted samples (Ag + Sr + He–SiC) were monitored using AFM in tapping mode before and after annealing. Samples were mounted on an aluminium stubby using double-sided tape with a diamond cantilever. Samples were analyzed using the *Bruker Dimension Icon* with ScanAsyst AFM system. AFM images were obtained with a scan size of 20 μm × 20 μm. *NanoScope* Analysis software was used to analyse AFM micrographs.

The elemental depth profiles of the implanted species were monitored using Rutherford Backscattering Spectrometry (RBS) and heavy ion Time-of-Flight Elastic Recoil Detection Analysis (ToF-ERDA) at iThemba LABS TAMS. The RBS analysis was using a 2 MeV α-beam provided by the 6 MV Pelletron Tandem accelerator. The backscattered α-particles were detected at a scattering angle of 150° using a solid-state detector. The charge collected per spectrum was set at 0.5 μC. ToF-ERDA was particularly used to monitor the migration of He, in addition to the other two implant species. The ERDA measurement made use of a 20 MeV ^197^Au^6+^ incident beam. A silicon PIPS® detector was used to measure the recoil ions' energy in coincidence with their time of flight, using two carbon foil-based timing detectors spaced 0.60 m apart. As a result of coincidence measurements, recoil particles are separated according to their atomic mass in a 2D ToF vs Energy scatter plot [27]. From this scatter plot elemental energy spectra are generated, and then an energy-to-depth conversion algorithm is used to calculate elemental depth profiles. The Potku data analysis program was used in this work to extract energy spectra and calculate elemental depth profiles [28].

## 3 Results and discussion

**Fig. 1:**
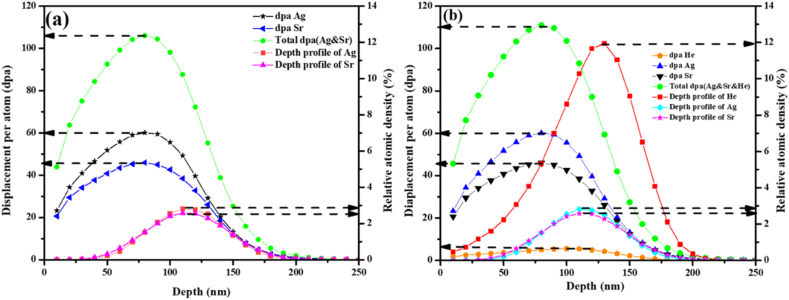
Simulated depth profiles and displacement per atom (dpa) of (a) Ag (360 keV) and Sr (280 keV) and (b) Ag (360 keV), Sr (280 keV), and He (17 keV) implanted into SiC.

Before implantation, simulations of ions to be implanted into SiC were performed. These were necessary in choosing the ions energy that will result in implanted species having approximately the same projected range, i.e., overlap. Fig. 1 shows the simulated depth profiles and displacement per atom (dpa) from the stopping and range of ions in matter SRIM 2010 [29] of 360 keV Ag, 280 keV Sr, and 17 keV He ions implanted into SiC. A density of 3.21 g/cm^3^ for SiC and displacement energies of 35 and 20 eV for silicon and carbon, respectively, were used in the simulations [30]. If one assumes that the critical amorphization of SiC is 0.3 dpa [31], all implantations will completely amorphized SiC at room temperature. However, our implantations were performed at temperatures well above the critical amorphization temperature of SiC [26,32]. Therefore, the samples were not expected to be fully amorphized under the implantation conditions used in this study. In Fig. 1 (a), Ag and Sr depth profiles overlap with a projected range around 120 nm below the surface. As shown in Fig. 1(a), individual implantation of Ag and Sr retained maximum lattice damage of 60 dpa and 45 dpa at a depth of 90 nm below the surface, respectively. The total lattice damage of 106 dpa at 85 nm was retained by both Ag and Sr ions. Helium ions retained a maximum damage of 5.3 dpa at a depth of 110 nm below the surface- Fig. 1 (b). Trible implantation of He, Ag and Sr retained a total maximum lattice damage of about 110 dpa at a depth of 85 nm depth below the surface. More importantly, the triple implantation resulted in the overlapping profiles of Ag, Sr and He which allows investigation on the effect of He in the migration behaviour of Ag and Sr co-implanted into SiC.

**Fig. 2:**
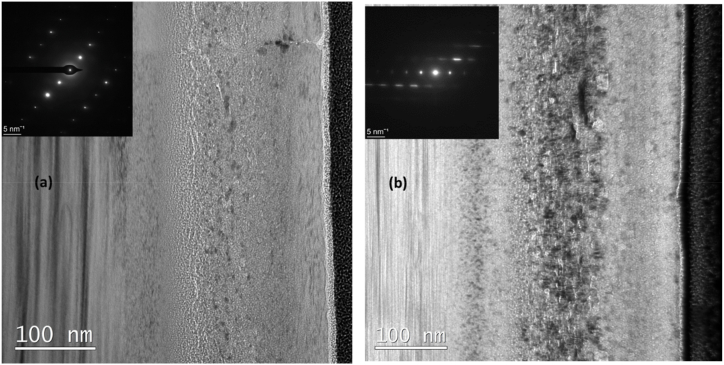
Bright field TEM micrographs of Ag + Sr + He–SiC sample before (a) and after annealing at 1000 °C for 5 h (a) together with their respective SAD pattern taken from the damage SiC.

Fig. 2 shows the under-focus bright field TEM (BFTEM) micrographs of the as-implanted (a) and annealed (b) Ag + Sr + He–SiC samples. Triple implanted SiC resulted in the appearance of brighter elongated contrast and darker contrast regions in the implanted SiC layer, as shown in Fig. 2(a). The brighter elongated contrast regions became mostly spherical and bigger after annealing at 1000 °C (see Fig. 2(b)). This was accompanied by the increase in density and size of the darker contrast regions. In the BF micrographs the darker regions might be Ag/Sr clusters in the triple implanted SiC while the brighter regions might be helium bubbles. The absence of brighter regions in the SiC co-implanted with both Ag and Sr as reported in Ref. [11] is a proof that the brighter regions are helium bubbles while the darker regions might be Ag or Sr precipitates. Ag precipitates were observed in SiC co-implanted with Ag and Sr [11]. As expected, due to elevated implantation temperatures, the selected area diffraction (SAD) patterns of the as-implanted Ag + Sr + He–SiC indicate the lack of amorphization in the implanted SiC layer.

**Fig. 3:**
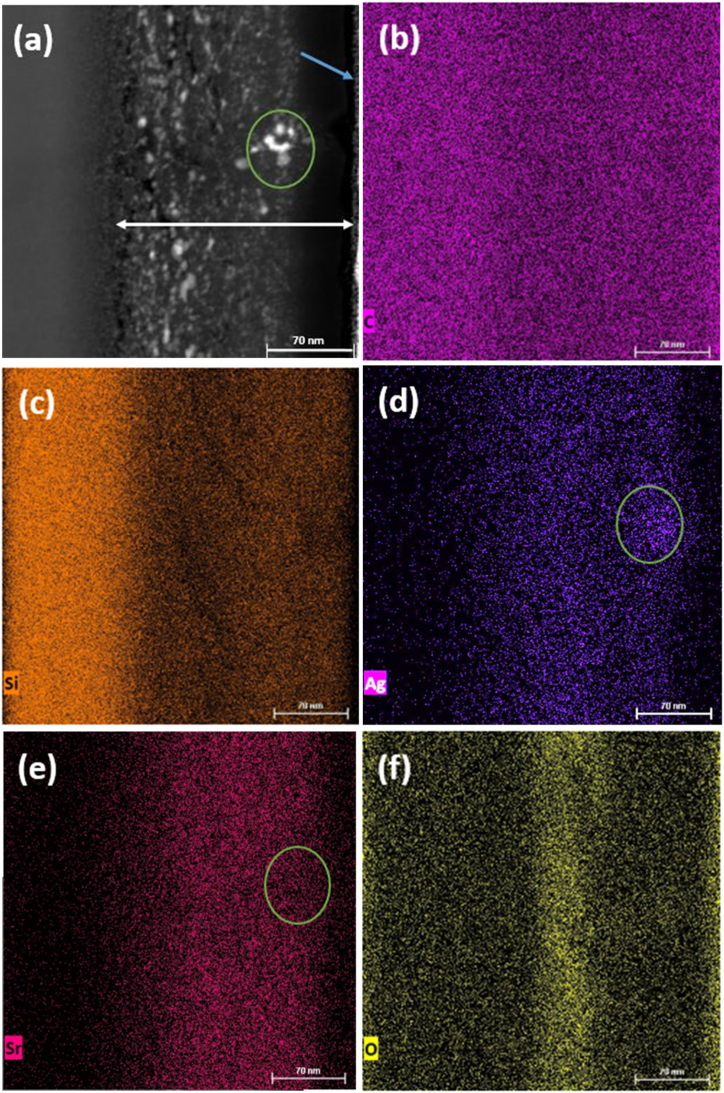
(a) High-angle annular dark-field (HAADF) scanning tunnelling electron microscopy (STEM) micrograph of the as-implanted Ag + Sr + He–SiC sample, and corresponding EDX mapping of (b) C, (c) Si, (d) Ag, (e) Sr, and (f) O elements in the damaged SiC layer. White double arrow shows the implanted region, while the blue arrow shows the surface, and the green circles shows Ag clusters.

**Fig. 4:**
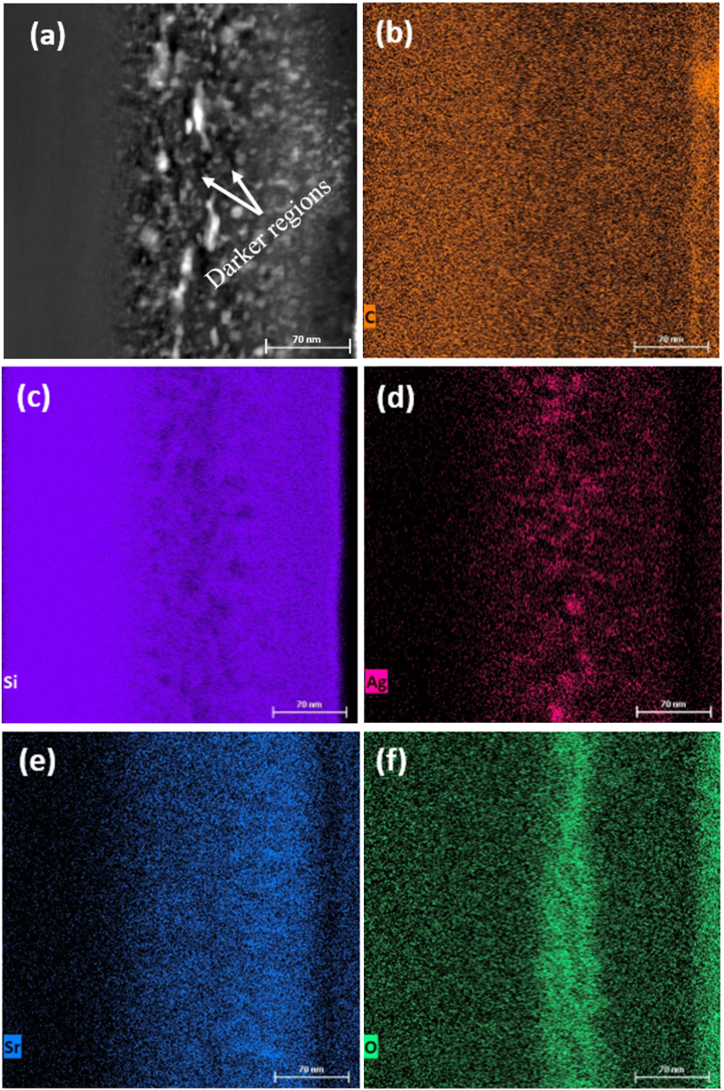
(a) High-angle annular dark-field (HAADF) STEM micrograph of Ag + Sr + He–SiC annealed at 1000 °C for 5 h and EDX mapping of (b) C, (c) Si, (d) Ag, (e) Sr, and (f) O.

Fig. 3, Fig. 4 show the high-angle annular dark-field (HAADF) scanning tunnelling electron microscopy (STEM) micrographs of the as-implanted Ag + Sr + He–SiC and annealed Ag + Sr + He–SiC samples respectively, together with energy dispersive X-ray (EDX) mapping of the elemental composition. The blue arrow indicates the surface while the white double arrow indicates the thickness of the damaged layer of SiC, which is about 200 nm from the surface. In HAADF micrograph the high contrast or brighter regions indicate high atomic number (Z) or heavier element and dark contrast indicates the low Z or lighter element. Therefore, the darker regions in Fig. 3 (a) might be helium bubbles or cavities and the brighter regions might be Ag or Sr clusters. HAADF micrographs of Ag and Sr individually implanted into SiC showed neither cavities nor bubbles but uniform distributed implants while HAADF micrograph of Sr and Ag co-implanted had Ag precipitates due to irradiation induce diffusion of Ag at 600 °C [11]. Therefore, without a doubt the darker regions are helium bubbles due to helium implanted into the defective SiC at 350 °C and the brighter regions are either Ag or Sr precipitates.

EDX mapping of elements is shown Fig. 3 (b)–(f). Carbon is uniformly distributed in the implanted layer while there is Si deficiency in the same damage layer (Fig. 3(b) and (c). Hence, triple implantation resulted in Si deficiency in damage layer where elongated He nano-bubbles were formed. The assertion that the white regions in Fig. 3 (a) are Ag precipitates and are formed in cavities is indicated by the green circles in Fig. 3(a), (d) and (f). The green circles indicate that the structures on the as-implanted sample close to the surface in Fig. 3(a) are Ag precipitates-Fig. 3 (d). These clusters are formed where there is Si deficiency close to the surface (Fig. 3(c)) while C atoms are uniformly distributed. Implantation of Sr in SiC at 600 °C may cause Ag to diffuse and precipitate as reported in Ref. [11]. Fig. 3(e) shows a uniform distribution of Sr. EDX mapping also confirmed the appearance of oxygen in the damaged layer. Surface oxygen in the implanted SiC as a result of the formation of native SiO_2_ on the surface has been reported [12]. In this study, as can be seen in Fig. 2 (f), the oxygen detected even within the damage SiC layer might be due to implantation of Sr and He ions causing the migration of oxygen into the damage layer.

Fig. 4 shows the HAADF STEM micrograph of Ag + Sr + He–SiC annealed at 1000 °C for 5 h, together with the EDX elemental mappings. The dark and white regions in Fig. 4(a) are He-nanobubbles and Ag precipitates, respectively. These He-nanobubbles are more spherical and have increased in size compared to the ones observed on the as-implanted Ag + Sr + He–SiC sample. This is due to Ag diffusion and agglomerating into silver precipitates trapped in cavities. The Si atoms seem to be nearly uniform distributed after annealing at 1000 °C indicating some limited recrystallization. However, some Si deficiency regions corresponding to the Ag precipitates can still be observed (see Fig. 4(c) and (d)). Carbon is still uniformly distributed in the annealed samples (Fig. 4 (b)). Some Sr-atoms seem to have diffused and distributed closer to the surface with a lateral layer where less Sr-atoms are found around the Ag precipitates-Fig. 4(e). Beyond this lateral layer the concentration slightly increases. The EDX mapping in Fig. 4(d) further confirms that the Ag precipitates became more visible at higher density suggesting that more silver is trapped in the cavities (see Fig. 4 (c)) as a result of the growth in their size. The EDX results also show that SiC retained oxygen after annealing at 1000 °C as shown in Fig. 4(f).

**Fig. 5:**
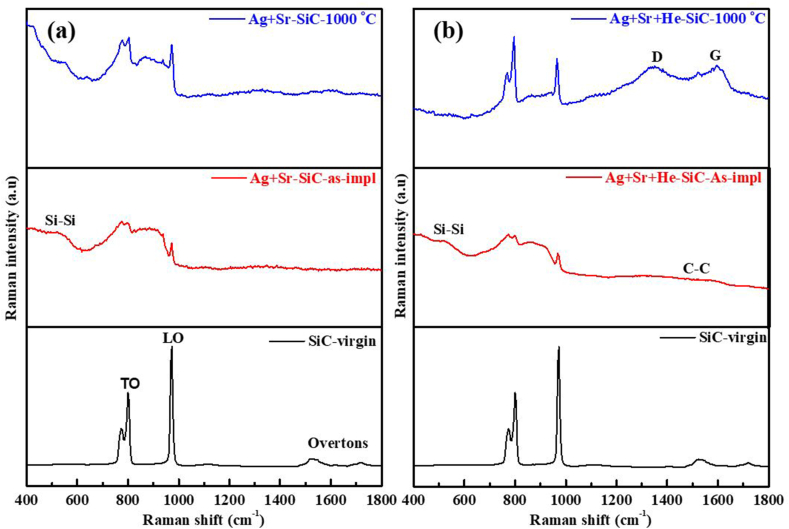
Raman spectra of (a) Ag + Sr–SiC and (b) Ag + Sr + He–SiC before and after annealing at 1000 °C. The Raman spectrum of un-implanted SiC is included for comparison.

**Fig. 6:**
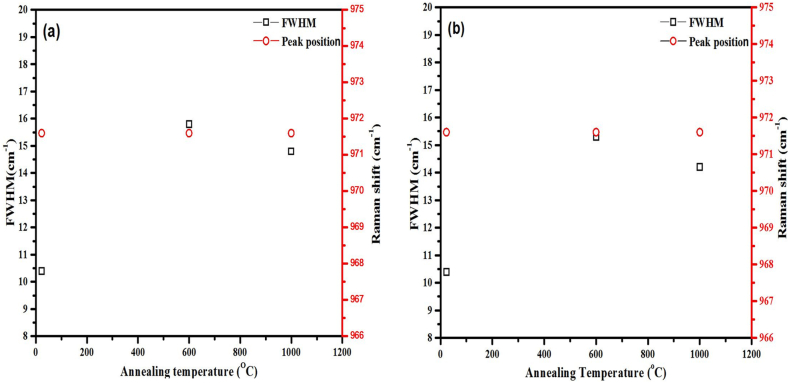
The peak position and FWHM of the LO peak as a function of temperature of (a) Ag + Sr–SiC and (b) Ag + Sr + He–SiC*.*

Fig. 5(a) and (b) show the Raman spectra of the as-implanted and implanted then annealed dual (Ag + Sr–SiC) and triple (Ag + Sr + He–SiC) implanted samples. Raman spectrum of the un-implanted SiC is included for comparison. The Raman peak position and FWHM of the LO mode as a function of annealing temperature are shown in Fig. 6. The Raman spectrum of the un-implanted sample has Raman characteristic peaks of SiC i.e., transverse mode (TO) which splits into two peaks around 765 cm^−1^ and 794.3 cm^−1^, the longitudinal mode (LO) peak around 971.6 cm^−1^ and two TO overtones peaks at 1516 cm^−1^ and 1715 cm^−1^ [12]. Both dual and triple implantation caused reduction in the characteristic peak intensities accompanied by peak-broadening with no shift in the LO peak position, indicating accumulation of defects or short-range disorder in the SiC structure. The accumulation of short-range disorder results in the formation of homonuclear bonds viz. Si–Si homonuclear band in the range 400–600 cm^−1^, and the C–C homonuclear band in the range 1100–1700 cm^−1^ [25,[33], [34], [35]]. The lack of amorphization is due to the implantation temperatures (600 °C and 350 °C) that are greater than the critical temperature of amorphization [26,32]. In both Raman spectra of dual and triple implanted SiC, the homonuclear bonds especially the Si–Si are dominant, which agrees with TEM result of the as-implanted sample in Fig. 3, where Si deficiency were observed while carbon was uniformly distributed. Annealing at 1000 °C caused a slight increase in LO mode intensity accompanied by the narrowing of the FWHM with no change in peak position in both the dual and triple implanted samples-Fig. 6 indicating limited recrystallization. Moreover, the decrease in Si–Si peak intensities were observed in both annealed samples. However, this was accompanied by the appearance of D and G carbon peaks in the annealed triple implanted sample. The appearance of D and G peaks in the annealed triple implanted sample is due to the presence of helium. Mokgadi et al. [25] observed similar phenomenon in the annealed SiC co-implanted with Sr and He at room temperature. It was found that Si and C interstitials migrate and agglomerate into clusters around He bubbles after annealing, which could be the reason for the intense G and D bands observed in the sample annealed at 1000 °C. In the annealed samples, the FWHM narrowed by 2.7 cm^−1^ in the Ag + Sr–SiC sample, while the FWHM of Ag + Sr + He–SiC sample narrowed by 1.1 cm^−1^. This indicates a high rate of vacancies-recombination or recrystallization in the Ag + Sr–SiC compared to the Ag + Sr + He–SiC samples. The reduced vacancy-recombination rate in the Ag + Sr + He–SiC sample might be due to the He exo-diffusion on the free surface at high temperatures which results in holes/craters (exfoliated blisters) in the implanted SiC layer that limits recovery. As shown in Fig. 6 (a) and (b), there is no observable difference in the LO mode position after implantation and after annealing suggesting minimum stretching and compression of the Si–C bonds. Comparing the Raman results of Ag + Sr–SiC and Ag + Sr + He–SiC samples, it is quite clear that the presence of He reduces recrystallization process. Similar He assisted recrystallization was reported in SiC co-implanted with He and Mg at RT annealed at 1573K [23].

**Fig. 7:**
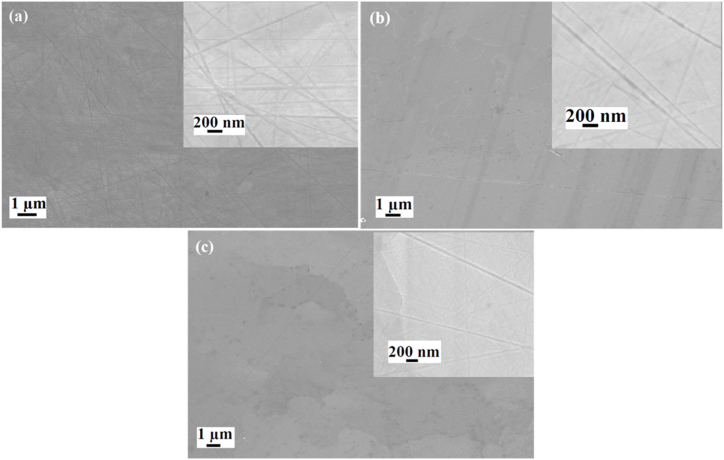
SEM micrographs of (a) un implanted SiC; (b) SiC co-implantation with Ag and Sr at 600 °C; and (c) SiC co-implanted with Ag and Sr and then annealed at 1000 °C for 5 h.

The SEM micrographs of the as-implanted Ag + Sr–SiC samples are shown in Fig. 7 together with SEM micrograph of the un-implanted SiC. The surface of the un-implanted SiC has polishing marks-Fig. 7 (a). These polishing marks are less visible in the dual as-implanted sample, as can be seen in Fig. 7(b). The reduction of polishing marks is due to the erosion caused by Ag and Sr bombardment on the surface and reduction in density caused by swelling of SiC [32]. The visibility of polishing marks indicates limited accumulation of short-range disorder which is consistent with the Raman results discussed earlier. After annealing, the polishing marks became less noticeable and decreased in number, as seen in the highmagnification inset of Fig. 7(c).

**Fig. 8:**
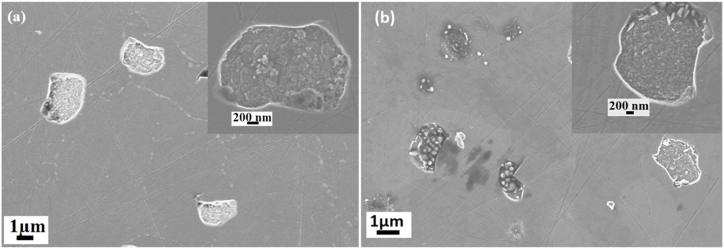
SEM micrographs of (a) as-implanted Ag + Sr + He–SiC and (b) annealed at 1000 °C*.*

Fig. 8 displays the SEM micrographs of Ag + Sr + He–SiC samples, before and after annealing at 1000 °C for 5 h. The SEM micrograph of the as-implanted sample, Fig. 8(a), reveals the presence of irregular structures on the surface with polishing marks still present. These irregular structures were not observed in the as-implanted Ag + Sr–SiC sample. Hence, they are due to the presence of He while the presence of polishing marks suggest lack of amorphization in agreement with TEM and Raman results. These irregular structures could be helium blisters or exfoliated blisters (holes). Annealing at 1000 °C caused an increase in the number of irregular structures on the surface, as can be seen in Fig. 8(b). Differentiating between blisters and craters using SEM is challenging; thus, further characterization of the Ag + Sr + He–SiC samples before and after annealing at 1000 °C was carried out using atomic force microscopy (AFM).

**Fig. 9:**
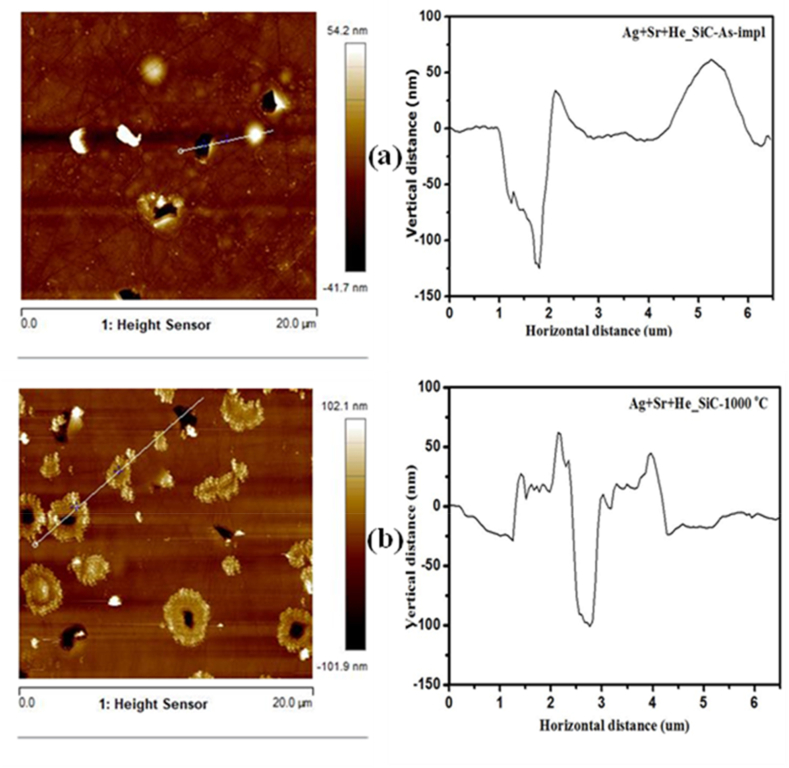
AFM 2D micrographs together with their line profiles of Ag + Sr + He–SiC (a) before and (b) after annealing at1000 °C. for 5 h (b).

Fig. 9 Shows the AFM micrographs of the Ag + Sr + He–SiC samples before (a) and after annealing (b). The as-implanted and annealed micrographs have some dark and light structures. The dark structures are exfoliated blisters/craters while the light structures are blisters, and the white particles are dust [25]. The dark structures seen in Fig. 9 resemble the irregular structures confirming that the irregular structures observed in the SEM micrograph are craters. Interstitials and vacancies are present in the Ag + Sr–SiC samples. In this study, He ions of 17 keV were implanted in the Ag + Sr–SiC samples. As the implantation was done at high temperatures, the vacancies were mobile which increased the probability of helium agglomerating to form bubbles. When bubbles form within the material, the high internal pressure in vacancies results in the deformation of the surface. Hence, the blisters appear on the surface. Due to more pressure within the largest blisters, or radial stresses exerted on the blisters [36,37], the surface of the blister erupt, leading to exfoliation during implantation at higher temperature and after annealing resulting in the appearance of craters on the surface. The out-diffusion of He implanted after annealing at higher temperatures resulting in the formation of cavities in the implanted SiC layers (Fig. 9(b)) has also been reported [12,38]. However, the appearance of exfoliated blisters in the as-implanted sample surface in Fig. 9 (a) might be indicating the out-diffusion of He during implantation at 350 °C.

**Fig. 10:**
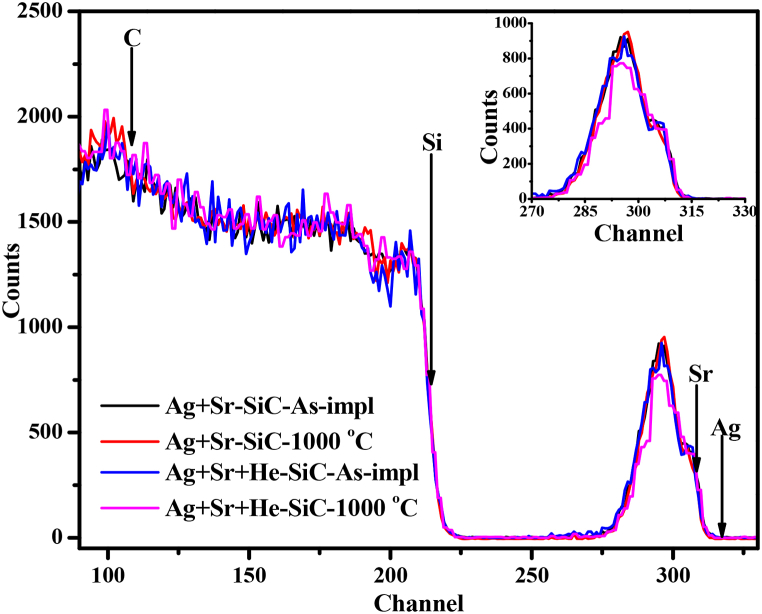
RBS spectra of the as-implanted and annealed Ag + Sr–SiC and Ag + Sr + He–SiC samples.

The migration of Ag and Sr implanted into SiC in the absence and presence of He were investigated before and after annealing at 1000 °C for 5 h was monitored by RBS. Fig. 10 shows the RBS spectra of the Ag + Sr–SiC and Ag + Sr + He–SiC samples before and annealing. The arrows in Fig. 10 indicate the surface positions of elements in energy channel numbers. The profiles of implants are asymmetric due to the overlapping of the implanted Ag and Sr and Ag forming precipitates or clusters as seen in Fig. 3 and reported Ag and Sr co-implanted SiC [11]. Implantation of He caused no change in the profile of the implanted indicating no detectable migration due to formation of He bubbles.

No change was observed in the Ag + Sr–SiC samples annealed at 1000 °C indicating no detectable migration of implanted Ag and Sr. These results are in agreement with the previously reported results of Ag and Sr co-implanted into SiC then at 1000 °C [11]. Annealing of Ag + Sr + He–SiC caused reduction in intensity accompanied by some slight movement towards the surface indicating some limited migration and loss. However, the inability of RBS in distinguishing between Ag and Sr makes it impossible to clearly identify which implanted species are migrating after annealing at this temperature. Hence, the Ag + Sr + He–SiC samples were further characterized by heavy ion ToF ERDA. Heavy ion ToF-ERDA has an added advantage over RBS due to its ability to detect He.

**Fig. 11:**
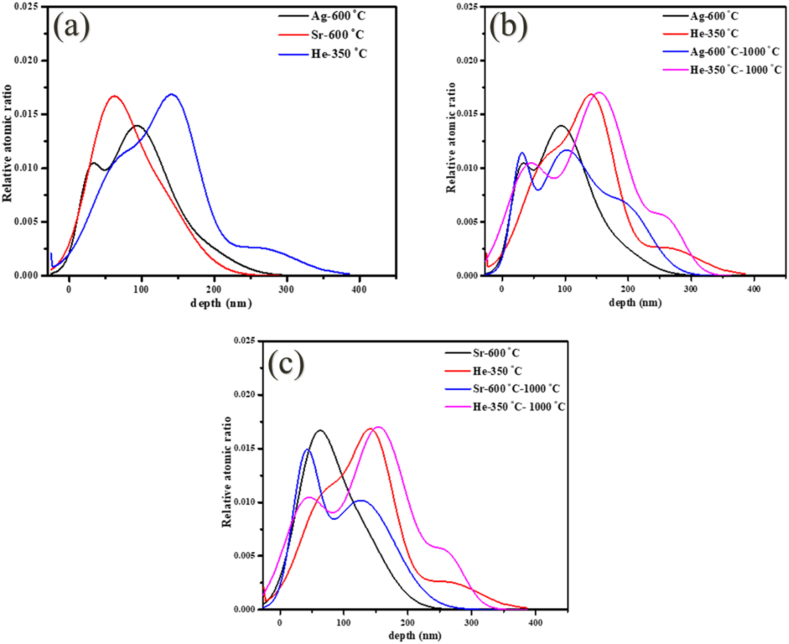
ERDA depth profiles of Ag, Sr and He obtained for the Ag + Sr + He–SiC before (a) and after annealing at 1000 °C for 5 h (b) and (c).

Fig. 11 shows ERDA depth profiles of Ag, Sr, and He from the Ag + Sr + He–SiC sample before (a) and after annealing (b) and (c). As predicted by SRIM in Fig. 1, the Ag, Sr, and He profiles are overlapping, allowing the investigation of the effect of He in the migration behaviour of Ag and Sr. As expected for implanted profiles, the as-implanted Sr depth profile was almost Gaussian and symmetric with peak position around 65 nm below the surface while the Ag profile consisted of the double peak profile i.e., a prominent peak at about 92 nm and another peak at 33 nm below the surface. The He profile was also asymmetrical with a prominent peak around 150 nm and two shoulders around 65 and 280 nm below the surface. The double peak profile of Ag in the Ag + Sr + He–SiC samples is due to silver forming precipitates during co-implantation of Sr and He. Similar double peak Ag profile was observed in as-implanted Ag + Sr–SiC samples [11] and is supported by the EDX elemental mapping of Ag in Fig. 3 (d). The first as-implanted peak of Ag is analogous to the Ag precipitates forming closer to the surface indicated by the green circle in Fig. 3 (d). Fig. 11 (b) shows that the depth profile of the He distribution is consistent with the STEM micrograph in Fig. 3 (a). The as-implanted Sr profile is uniformly distributed, which agrees with STEM of Fig. 3(e). Comparing these results with the as-implanted results of Ag + Sr–SiC in Ref. [11] and SRIM results in Fig. 1, it is evident that co-implantation did cause some migration of pre-implanted Ag and Sr accompanied by some loss from the surface. This loss seems to be more pronounced for Sr as compared to Ag. The migration might be due to vacancy migration during He implantation which also results in the formation of He elongated nano-bubbles, while the formation of blisters results in the formation of cavities in the damaged SiC layer which may assist in the trapping of the implanted species. Based the as implanted results in Fig. 11 (a) and TEM, STEM and EDX results, Ag is the most trapped species in the cavities. Similar trapping of Ag by He cavities has been reported in Ag and He co-implanted into SiC at room temperature then annealed at 1100 °C for 5 h [12] while Sr trapping was also reported in the Sr and He co-implanted SiC annealed at 1000 °C for 5 h [24]. The lack of or insignificant trapping of Sr by cavities in the present study might be due to unavailability of empty cavities as they are already occupied by Ag precipitates as seen in Fig. 3.

Annealing at 1000 °C resulted in Ag profile with main peak at 103 nm and other peaks at 33 nm and 200 nm below the surface indicating the Ag clustered at different regions. These clusters are areas of with Ag precipitates as seen in Fig. 4 (a) and (d). Sr profile became a double peak after annealing at 1000 °C with peaks at 42 and 128 nm below the surface indicating clustering of Sr in these two regions while the He profile did not change much, except for moving towards the surface. Like with Ag clustering, He-profile also show similar clustering which is the coalescence into He-bubbles below the surface. This agrees with TEM results in Fig. 2, Fig. 4 where He-bubbles were seen to be randomly distributed and the concentration decreasing close to the Ag precipitates. This decrease could be due to formation of cavities as a result of He-*exo*-diffusion. Likewise, in the study by Li et al. [22] Fe atoms were found to be trapped in cavities and forming clusters within them. Similar trapping was also observed for Ag co-implntated with He at RT after annealing at 1100 °C for 5 h [12] and for Ag and He co-implanted into SiC after annealing at 1000 °C for 5 h [25].

The Sr-profile after annealing has a high concentration of Sr-atoms found closer to the surface and this concentration decreasing around the 80 nm, which is around the Ag precipitates or He-cavities. This agrees very well with EDX elemental mapping in Fig. 4 (e). What is also evident in the depth profiles of Ag, Sr, and He of the annealed samples (see Fig. S1 in the supplementary data) is that the peaks of the Sr double peak are in the region with less Ag or in the minimum of Ag peak indicating that they might be trapped by the new cavities formed due to the migration of He. Since the migration of implanted species was not seen in the Ag and Sr co-implanted samples after annealing at 1000 °C, it is clear that He bubbles enhance migration while cavities trap the implanted species in the triple implanted SiC.

## 4 Summary

In this study, the effects of helium, strontium, and silver triple ions implanted into SiC were investigated. Ag ions were first implanted into polycrystalline SiC at 600 °C, followed by implantation of Sr ions also at 600 °C. Some of the co-implanted samples were then implanted with He ions at 350 °C. The dual (Ag + Sr–SiC) and triple (Ag + Sr + He–SiC) implanted samples were annealed at temperature at 1000 °C for 5 h. Both dual and triple implantation caused accumulation of defects without amorphization of the SiC structure. Implantation of Sr in the dual implanted sample caused the formation of Ag precipitates indicating some migration of pre-implanted Ag during the co-implantation of Sr. Implantation of He in the triple implanted SiC resulted in formation of Ag participates accompanied the formation of He bubbles and cavities in the implanted SiC layer. Some limited migration of pre-implanted Ag and Sr towards the surface was also observed in the triples implanted samples. Moreover, blisters and craters on the surface indicating both formation of bubbles and exo-diffusion of He-atoms from He-bubbles below the surface were observed in the triple implanted samples. The exfoliation of the surface increased after annealing resulting in more craters on the surface. Some limited recrystallization was observed in both annealed dual and triple implanted samples. No migration of implanted species was observed in the Ag + Sr–SiC samples annealed at 1000 °C while migration of Ag and Sr was observed in the Ag + Sr + He–SiC samples annealed at the same temperature. The migration of Ag in the annealed triple implanted SiC was governed by their trapping of Ag in the He cavities within the implanted SiC layer. The cavity trapping favours only Ag-atoms to the exclusion of Sr-atoms. Therefore, He bubbles enhance migration while cavities trap the implanted species. It is important to note that the findings of this study are closely related to what is occurring in SiC in the modern fission nuclear reactor fuel particle. Under normal conditions of operation or accident, the SiC layer is exposed to a various fission products including Ag and Sr in the presence of He. Hence, these results are vital for the safety of modern nuclear reactors.

## Author contribution statement

Gcobani Ntshobeni: Performed the experiments; Analyzed and interpreted the data; Wrote the paper.

Zaki Abdalla: Conceived and designed the experiments; Analyzed and interpreted the data; Wrote the paper.

Thapelo Mokgadi: Performed the experiments.

Mbuso Mlambo: Conceived and designed the experiments.

Eric NJOROGE: Analyzed and interpreted the data.

Mandla Msimanga; Alexander Sohatsky; Vladimir Skuratov: Contributed reagents, materials, analysis tools or data.

Thulani Hlatshwayo: Conceived and designed the experiments; Analyzed and interpreted the data.

## Data availability statement

Data will be made available on request.

## Declaration of competing interest

The authors declare no conflicts of interest.
