# Supplementary figures and images for: The effects of helium, strontium, and silver triple ions implanted into SiC
Source: Heliyon. 2023 Oct 10;9(10):e20877. doi: 10.1016/j.heliyon.2023.e20877 (PMC10585298; doi:10.1016/j.heliyon.2023.e20877)

***Supplementary data***

**
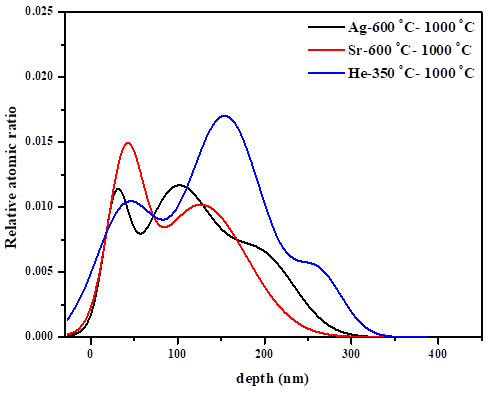
**

***Fig. S1*.***Shows the depth profiles of Ag, Sr, and He of the annealed samples.*

Supplement: Multimedia component 1 [file mmc1.docx]
